# Increased maternal nighttime cortisol concentrations in late gestation alter glucose and insulin in the neonatal lamb

**DOI:** 10.14814/phy2.12548

**Published:** 2015-09-14

**Authors:** Andrew Antolic, Xiaodi Feng, Charles E Wood, Elaine M Richards, Maureen Keller-Wood

**Affiliations:** 1 Department of Pharmacodynamics, University of FloridaGainesville, Florida; 2 Department of Physiology and Functional Genomics, University of FloridaGainesville, Florida

**Keywords:** Cortisol, glucose, insulin, neonate

## Abstract

Previous studies in our laboratory have shown that a modest chronic increase in maternal cortisol concentrations impairs maternal glucose metabolism and increases the incidence of perinatal stillbirth. The dramatic outcomes prevented our ability to study the effects of maternal hypercortisolemia on neonatal growth, glucose metabolism, and hypothalamo–pituitary–adrenal axis response. Therefore, we developed a model in which pregnant ewes are infused for 12 h/day at 0.5 mg·kg^–1^·day^–1^ from day 115 of gestation until delivery (˜145), elevating nighttime plasma cortisol concentrations. This pattern of elevation of cortisol mimics that in patients with elevated evening cortisol concentrations, as in Cushing’s syndrome or chronic depression. Plasma cortisol, glucose, insulin, and electrolytes were measured during pregnancy and postpartum in control and cortisol-infused ewes and their postnatal lambs for the first 14 days after delivery. Neonatal growth and plasma ACTH, aldosterone, renin activity, and electrolytes, and organ weights at 14 days of age were also measured. Infusion of cortisol increased maternal plasma cortisol during pregnancy but not postpartum, and did not alter neonatal ACTH or cortisol. Although maternal glucose and insulin concentrations were not changed by the maternal infusion of cortisol, neonatal plasma glucose was increased and plasma insulin was decreased compared to those in the control group. Neonatal ponderal index and kidney weight were reduced, left ventricular wall thickness was increased, and plasma sodium and creatinine were increased after maternal cortisol infusion. These results suggest that excess maternal cortisol concentrations in late gestation alter growth, glucose and insulin regulation, and organ maturation in the neonate.

## Introduction

A growing body of clinical, experimental, and epidemiological evidence suggests that chronic maternal stress during pregnancy is a major risk factor for poor obstetric outcomes (Copper et al. 1996; Davis and Sandman 2010). Activation of the hypothalamo–pituitary–adrenal (HPA) axis by psychological or physiological stressors induces the synthesis and release of the glucocorticoid hormone cortisol. Cortisol’s actions include the transrepression and activation of genes involved in adaptive mechanisms to stress, such as the stimulation of glucose production and support of the cardiovascular system; however, prolonged or excessive exposure is associated with deleterious effects in cells and tissues (Moghadam-Kia and Werth 2010). Throughout pregnancy, maternal cortisol concentrations rise, producing a period of physiological hypercortisolemia. The increased maternal cortisol facilitates delivery of nutrients to the developing fetus (Butte 2000). Placental 11β-hydroxysteroid-dehydrogenase-2 (11β-HSD-2) (Yang et al. 1997) acts as a partial buffer through conversion of cortisol to cortisone. As a consequence, it is estimated that about 2% of maternal cortisol crosses the placenta to the fetus; however, because of the differences in volume of distribution for cortisol, this represents nearly all of the fetal plasma cortisol concentration until very close to term when fetal adrenal production of cortisol increases (Dixon et al. 1970; Hennessy et al. 1982). Synthetic glucocorticoids are able to bypass this degradation, but maternal hypersecretion of cortisol also results in increased cortisol exposure to the fetus. Although the normal increase in fetal cortisol production near term is essential for organ maturation (Liggins 1994), overexposure of the developing fetus to maternal glucocorticoids has been proposed as a mechanism for fetal programming (Moisiadis and Matthews 2014). Increased exposure to maternal glucocorticoids has primarily been studied in the context of antenatal glucocorticoid therapy, rather than increased maternal secretion of the endogenous adrenal corticosteroid, cortisol. In humans and in most animal species, glucocorticoid exposure increases the incidence of decreased body weights at birth or produces small for gestational age offspring (Reinisch et al. 1978; Barrada et al. 1980; Johnson et al. 1981; Benediktsson et al. 1993; Newnham and Moss 2001). Antenatal glucocorticoid therapy also alters HPA responses in the newborn (Waffarn and Davis 2012) and has been implicated in adverse cardiac and renal growth (Bensley et al. 2010).

Little is known about effects of increased maternal cortisol secretion near term on early postnatal growth and metabolism. In order to investigate the effects of chronic exposure to increased maternal cortisol on neonatal growth, metabolism, and organ maturation, we developed a model of chronic stress during pregnancy in which the ewe is infused with cortisol for 12 h overnight beginning at gestational day 115 (term is ˜145 days). In previous studies, we had found that chronic continuous infusion of cortisol to the ewe not only resulted in chronic increase in maternal glucose, but also resulted in an increased incidence of peripartum fetal death (Keller-Wood et al. 2014). Therefore, a model using 12 h of cortisol infusion, producing half the daily total dose, but similar increase in nighttime plasma cortisol concentrations, was used in the present study. This method mimics conditions similar to those seen in patients with Cushing’s syndrome and chronic depression, in which there is a loss of the normal circadian rhythm of cortisol, resulting in elevated nighttime cortisol concentrations (Laudat et al. 1988; Raff et al. 1998; Scharnholz et al. 2010). We hypothesized that increased cortisol exposure in utero and withdrawal from the excess exposure at birth would result in an impaired HPA reactivity in the neonate and decreased neonatal growth, with impaired glucose metabolism in the early postnatal period.

## Methods

Two groups of black-faced ewes and their lambs were studied. All animals were housed in a facility with light (lights on 0700 to 1900) and temperature controlled rooms throughout the study period; all animal use was approved by the University of Florida Institutional Animal Care and Use Committee. Ewes were fed a diet of pelleted feed according to NRC standards for the ewe’s body weight and gestation; feeding was further supplemented with alfalfa hay beginning at day 140 of gestation. Ewes were assigned to one of two groups of ewes at 115 days: a control group (*n *=* *7; 6 singleton and 1 twin pregnancies) with no infusion, and a group of ewes treated with cortisol (0.5 mg/kg/day infused from 2100 to 0900, infusion rate 1.4 mL/h, *n *=* *8; 7 singleton and 1 twin pregnancies). The two groups of ewe had similar weights at surgery (control: 92 ± 5 kg; cortisol: 82 ± 5 kg) and body condition scores at term (control: 3.2 ± 0.1; cortisol 2.9 ± 0.1).

At approximately day 115 (±1) of gestation, surgery was performed under isoflurane anesthesia. A flow probe (6 mm 6PSS; Transonics Inc., Ithaca, NY) was placed on the main uterine artery. Catheters were also placed in both maternal femoral arteries and veins; the uterus was not opened and the fetuses were not instrumented. Ewes were treated at the end of surgery and for 2 days postoperatively with flunixin meglamine (1 mg/kg; Merck Animal Health, Germany) as an analgesic, and were treated with antibiotic (Polyflex, Boehringer Ingelheim Vetmedica, Inc., St. Joseph, MO) for 3–5 days postoperatively. Rectal temperature was measured twice a day for 5 days.

A programmable timer was used to control the infusion pump (Chronotrol, ChronTrol Corporation, San Diego, CA). This pattern of cortisol infusion was designed to mimic the effect of Cushing’s disease in humans, which results in higher than normal evening concentrations of cortisol. Although sheep do not have a circadian rhythm in cortisol (Bell et al. 1991), the activity associated with normal husbandry and feeding, which occurs in the morning after lights on, would be expected to increase morning cortisol concentrations in the ewes, and since most ewes finish the feed before the timing of feeding (just after lights on), this would also be expected to increase maternal cortisol near the time of refeeding each morning (Simonetta et al. 1991). The timing of the cortisol infusion was designed to allow adequate time after lights on to allow for collection of blood sampling and measurement of uterine blood flow while the infusion is still ongoing.

Blood samples (8 mL) were collected on approximately 125, 130, 135, and 140 days of gestation and on day 145 if the lamb had not yet been delivered. Blood samples were analyzed for maternal electrolytes (Roche Electrolyte analyzer 9180), glucose and lactate (YSI Model 2700 glucose/lactate analyzer, Yellow Springs, OH), cortisol (Siemens Coat-a Count kit; Los Angeles, CA), and insulin (Alpco ovine insulin kit, Alpco Diagnostics, Salem, NH) as described previously (Keller-Wood et al. 2014). After the blood sample was collected, uterine blood flow was measured for at least 30 min using an ambulatory Bluetooth-based acquisition system (Transonics Physiogear; Transonics Inc., Ithaca, NY). A glucose tolerance test was also performed in each ewe at approximately day 133 of gestation (0.4 g/kg i.v.) as described previously (Feng et al. 2013).

In both groups one ewe was euthanized in labor due to dystocia, and two lambs were stillborn in the cortisol-exposed group. One set of twins was born in each group of ewes. A total of four male and four female lambs were born to seven control ewes at 145 ± 1 days (range 144–149 days) gestation, and four male and five female lambs were born to eight cortisol-infused ewes at 145 ± 1 days (range 142–146 days) gestation.

The lambs were sampled after birth (after the ewe had licked off the lamb and the lamb was standing; control: 1.4 ± 0.5 h; cortisol: 1.6 ± 0.5 h, and subsequently at approximately 12, 24, 36, 48 h after birth and on postnatal days 3, 6, 9, 12, and 14 for measurement of plasma cortisol, insulin, glucose, and lactate concentrations and packed cell volume). Plasma electrolyte, plasma protein, and hematocrit were also measured at birth and days 3, 6, 9, 12, and 14 after birth. Plasma ACTH (Bell et al. 1991) and plasma renin activity (Wood et al. 1990) were measured on days 0, 3, 6, 9, 12, and 14 after birth; aldosterone (Aldosterone EIA 501090; Cayman Chemical, Ann Arbor, MI) was measured in samples collected on days 0, 3, 6, and 14 days; and plasma creatinine (Calorimetric Assay kit 700460; Cayman Chemical) was measured in samples collected on days 0, 3, 6, 9, and 14 days; all samples in the study were analyzed at the same time to eliminate between assay variability. The sample near the time of birth was collected by percutaneous venipuncture of the jugular; subsequently an indwelling venous catheter (Intracath, 17 g needle and 19G catheter; The Deseret Company, Sandy, UT) was placed and used for sampling and for injections of glucose or insulin. Body weight and temperature was also measured daily. On postnatal days 3–5 and on days 10–12 glucose tolerance tests (0.25 g/kg) were performed; blood samples were collected at –5, 0, 2, 5, 10, 20, 30, 40, 50, 60, and 90 min after injection of glucose to determine the plasma insulin response to increases in plasma glucose. On postnatal days 5–8 an insulin tolerance test (0.25 U/kg) was performed; samples were collected at –5, 0, 5, 10, 15, 20, 30, 40, 50, 60, and 90 min after insulin injection to determine the plasma ACTH and cortisol response to decreased plasma glucose concentration. Plasma glucose and lactate concentrations were measured in all samples collected after both the glucose or insulin tolerance test.

Crown-to-rump and hock-to-hoof length of the rear legs were measured on days 0, 7, and 14. On days 14–15 after birth, ewes and their lambs were killed (Euthasol; Fort Worth, TX) and measurements of the weights of the heart, liver, adrenal, lung, kidneys, perirenal fat, pancreas, and brain were performed.

### Statistical analyses

Between-group differences in plasma hormone, glucose, lactate, and electrolyte concentrations over age in late gestation ewes, postpartum ewes, or postnatal lambs were compared by two-way analysis of variance (ANOVA) corrected for repeated measures across time (SPSS, IBM Corp., Armonk, NY). The effect of the sex of the lamb on these variables was analyzed using three-way ANOVA, also corrected for repeated measures over time. Responses to glucose in the pregnant ewes or to insulin or glucose injection in the postnatal lambs were also analyzed by two-way ANOVA. The maternal glucose responses were also fit to a 5-parameter fit [*y* = *y*0 + *a*(exp – *bt*) + *c*(exp – *dt*)] and the neonatal glucose responses were fit to a 4-parameter fit [*y* = *a*(exp – *bt*) + *c*(exp – *dt*)] as the samples in the lamb were only collected until 90 min in order to minimize the total sampled volume and therefore the estimates of baseline glucose at the end of the test were not possible to fit. The parameters were compared between the two groups by Student’s *t*-test. Differences in tissue weights between the two groups of lambs were also analyzed by *t*-tests.

## Results

### Effects of cortisol exposure in pregnancy on the pregnant and postpartum ewe

**Figure 1:**
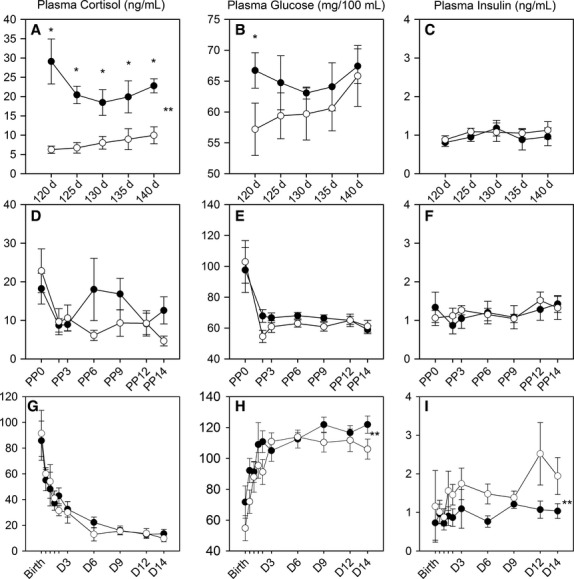
Plasma cortisol (A, D, G), glucose (B, E, H), and insulin (C, F, I) concentrations in studies in control ewes (white circles) or ewes that were infused with cortisol during pregnancy (0.5 mg·kg^–1^·day^–1^ for 12 h) (black circles). Values are shown for ewes during pregnancy (A–C) or in the postpartum period (D–F), and for their postnatal lambs (G–I). **A significant overall effect of maternal cortisol treatment. *Significantly different between groups at the indicated time point.

**Figure 2:**
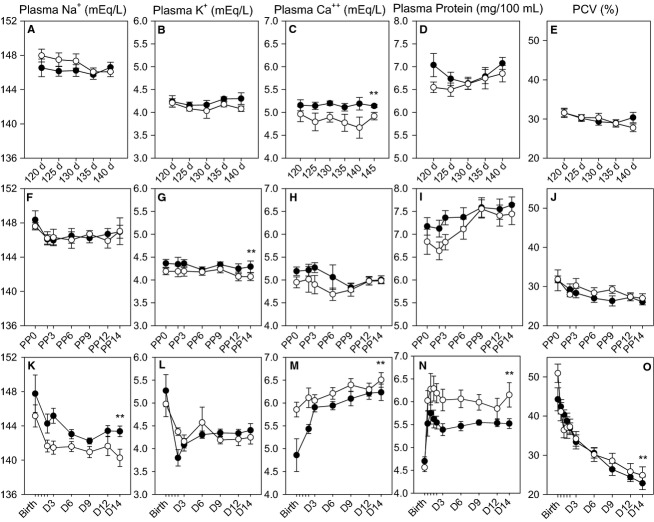
Plasma sodium (A, F, K), potassium (B, G, L), calcium (C, H, M), protein (D, I, N), and packed cell volume (PCV) (E, J, O) concentrations in studies in control ewes (white circles) or ewes that were infused with cortisol during pregnancy (0.5 mg·kg^–1^·day^–1^ for 12 h) (black circles). Values are shown for ewes during pregnancy (A–E) or in the postpartum period (F–J), and for their lambs during the postnatal period (K–O). **A significant overall effect of maternal cortisol treatment.

**Figure 3:**
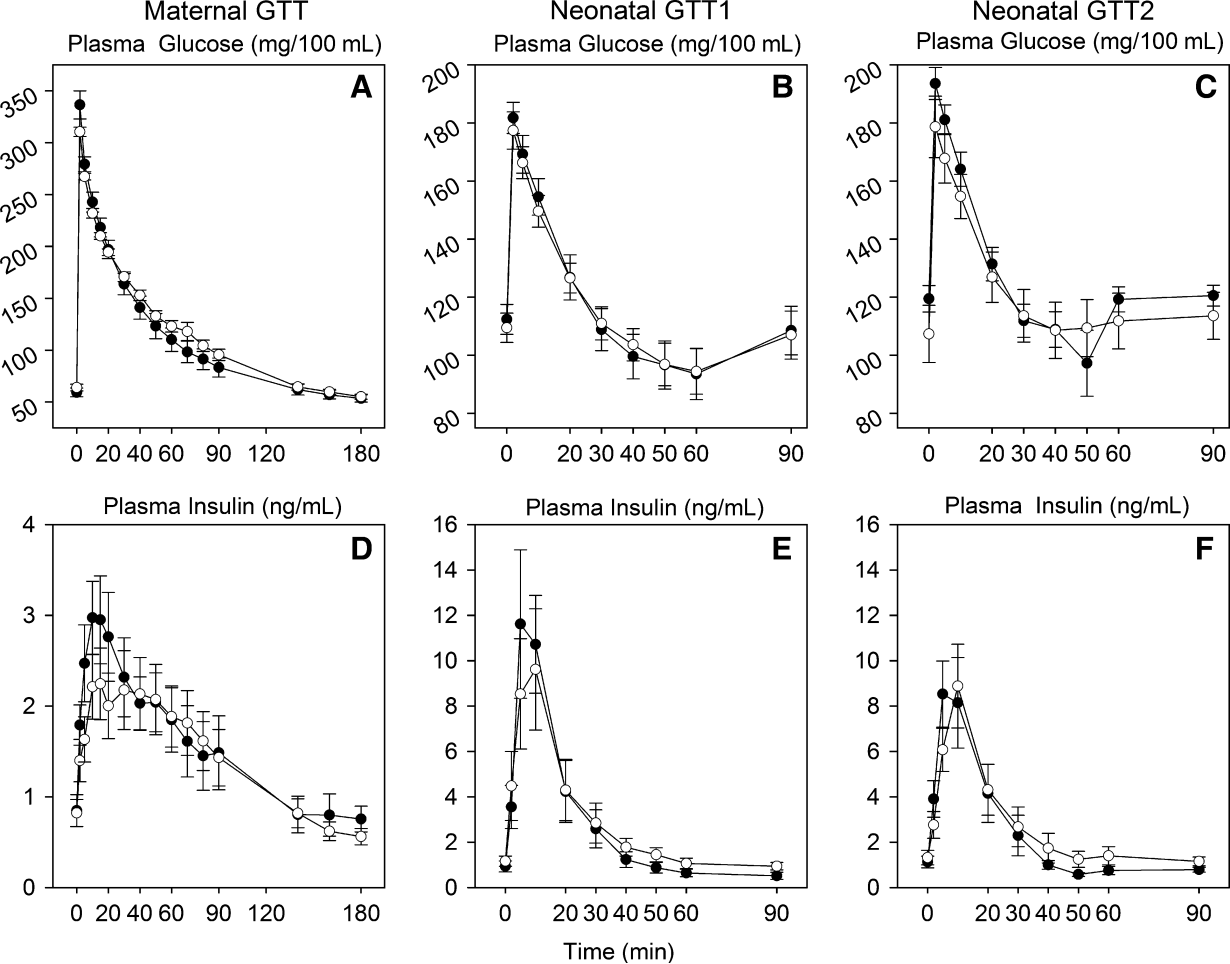
Plasma glucose (A–C) and plasma insulin (D–F) concentrations in response to intravenous injection of 0.40 g glucose/kg (maternal GTT; A, D) in control ewes (white circles) or ewes infused overnight with cortisol (0.5 mg·kg^–1^·day^–1^) (black circles) and in response to intravenous injection of 0.25 g glucose/kg in their postnatal lambs at 5–7 days of age (neonatal GTT1; B, E) or 10–12 days of age (neonatal GTT2; C, F).

As expected, the infusion of cortisol increased plasma cortisol concentrations during the infusion period in the treated ewes (Fig. 1A). Plasma glucose concentrations were not significantly increased in the ewes during the infusion of cortisol, except at the first time point (120 days, after 5 days of cortisol infusion; Fig. 1B). There were no effects of cortisol on plasma insulin, sodium, potassium, protein, or lactate concentration, or packed cell volume (PCV) (Figs. 1C, 2A, B, D, E; lactate not shown). PCV significantly decreased with gestational age (Fig. 2E). Plasma Ca^2+^ concentrations were significantly increased in the cortisol-infused group at days 130–140 of gestation (Fig. 2C). During the maternal glucose tolerance test, although there was no overall treatment effect, there was a significant effect of cortisol administration on both the glucose and insulin responses over time (Fig. 3A and D). When the glucose response was fit to a 5-parameter fit (*y* = *y*0 + *a* * exp – *bt* + *c* *exp – *dt*), the *a* parameter was significantly greater in the cortisol-treated ewes, indicating that the glucose distribution volume is reduced in the cortisol-treated ewes (Table 1).

**Table 1:** Glucose tolerance tests: results of 5-parameter fit (ewes) or 4-parameter fit (lambs) of glucose disappearance curve from 2 to 180 min (ewe) or 2 to 90 min (lamb)

	Pregnant ewes 5-parameter fit: *y* = *y*0 + (*a* ^*^ exp – bt) + (*c* ^*^ exp – dt)		Lamb 4-parameter fit *y* = + (*a* ^*^ exp – *bt*) + (*c* ^*^ exp – *dt*)			
			5–7 days		10–12 days	
	Control	Cortisol	Control	Cortisol	Control	Cortisol
*y*0	40.4 ± 4.4	42.1 ± 6.2				
*a*	112 ± 9	150 ± 9*	72 ± 13	104 ± 8*	85 ± 4	106 ± 3*
*b*	0.294 ± 0.075	0.438 ± 0.127	0.079 ± 0.014	0.056 ± 0.008	0.076 ± 0.008	0.075 ± 0.010
*c*	235 ± 15	220 ± 9	113 ± 13	90 ± 10	113 ± 8	109 ± 4
*d*	0.0205 ± 0.0035	0.0173 ± 0.0025	0.0033 ± 0.0017	.0010 ± 0.0010	0.0001 ± 0.0001	0.0001 ± 0.0001

* Significant difference in values between cortisol and control group (*P *<* *0.05, one-tailed Student’s *t*-test).

There were no differences in maternal cortisol, glucose, or insulin after delivery, when the cortisol infusion was terminated (Fig. 1D–F). However, the plasma potassium concentration was significantly greater in the postpartum ewes that had previously received the overnight cortisol infusion (Fig. 2G). Plasma Ca^2+^ significantly decreased after delivery; however, this decrease over time was attenuated in ewes that had previously been treated with cortisol during pregnancy (Fig. 2H). Plasma Na^*+*^, protein, and PCV were not significantly different postpartum in ewes that had previously received overnight cortisol treatment as compared to those that were untreated during pregnancy (Fig. 2F, I, J). Plasma glucose, lactate, and PCV decreased and plasma protein increased after delivery in both groups (Figs. 1E, 2J; lactate data not shown).

### Effects of maternal cortisol on neonatal growth and organ weights

Maternal cortisol infusion also did not alter body temperature or body weight at birth, or the change in body weight or temperature over the first 14 days of life (Table 2); there were no significant effects of sex of the lamb on either of these variables. Ponderal index was reduced overall in the lambs of cortisol-treated ewes (Table 2), although this effect of time was restricted to the male fetuses. There was a significant effect of sex on the change in ponderal index with postnatal age. In male lambs, the ponderal index at birth was reduced in the cortisol group, and in the females the ponderal index was significantly reduced in the cortisol group at day 14.

**Table 2:** Postnatal measures of growth and body temperature in control and cortisol-exposed lambs

	Body weight (g)**		Crown to rump (cm)**		Ponderal index (g/cm^3^)**						Body temperature (°F)**	
					Control			Cortisol*				
Age	Control	Cortisol	Control	Cortisol	(All)	(M)	(F)	(All)	(M)**	(F)	Control	Cortisol
Birth	5178 ± 589	5471 ± 290	54.2 ± 1.6	56.6 ± 1.8	0.031 ± 0.003	0.038 ± 0.004	0.025 ± 0.003	0.031 ± 0.003	0.025 ± 0.001*	0.035 ± 0.003	102.0 ± 0.3	102.5 ± 0.5
D3	6165 ± 615	6383 ± 383	–	–	–	–	–	–	–	–	103.1 ± 0.1	102.7 ± 0.3
D6	7181 ± 593	7371 ± 454	–	–	–	–	–	–	–	–	103.1 ± 0.2	103.2 ± 0.2
D7	7580 ± 631	7781 ± 501	62.6 ± 3.3	66.5 ± 17	0.032 ± 0.003	0.032 ± 0.003	0.031 ± 0.005	0.026 ± 0.001	0.026 ± 0.002	0.028 ± 0.001	102.9 ± 0.2	102.8 ± 0.2
D9	8180 ± 614	8481 ± 525	–	–	–	–	–	–	–	–	103.3 ± 0.3	103.2 ± 0.2
D12	9106 ± 666	9436 ± 578	–	–	–	–	–	–	–	–	103.7 ± 0.4	103.2 ± 0.2
D14	9690 ± 702	10,185 ± 618	71.3 ± 1.9	75.1 ± 1.6	0.026 ± 0.001	0.026 ± 0.001	0.027 ± 0.003	0.025 ± 0.000	0.026 ± 0.000	0.024 ± 0.000*	103.0 ± 0.2	102.9 ± 0.1

* Significant effect of maternal treatment with cortisol.

** Significant effect of postnatal age.

At postnatal day 14, organ weights were similar in the two groups of lambs (Table 3), with the exception of average kidney weight per gram body weight, which was significantly lower in the lambs after previous maternal cortisol infusion. Although the relative heart weight-to-body weight ratio was not different between the two groups, the left ventricular wall thickness was increased in the lambs exposed to higher in utero cortisol concentrations (control: 6.5 ± 0.3 mm; cortisol: 7.4 ± 0.3 mm), and this effect was also significant when the wall thickness was corrected for tibial length (Table 2). In contrast, right ventricular free wall thickness (control: 3.5 ± 0.3 mm; cortisol: 3.8 ± 0.1 mm) and septal thicknesses (control: 7.5 ± 0.5 mm; cortisol: 7.9 ± 0.3 mm) were not significantly increased in these lambs. There was no overall effect of sex of the lamb on any of the organ weights or measures of cardiac size.

**Table 3:** Relative organ weights and cardiac wall thicknesses at necropsy in control and cortisol exposed lambs

	Control	Cortisol
Brain/body weight (×10^2^)	0.691 ± 0.044	0.662 ± 0.042
Lung/body weight (×10^2^)	2.34 ± 0.15	1.95 ± 0.16
Liver/body weight (×10^2^)	2.85 ± 0.13	2.60 ± 0.08
Kidney/body weight (×10^2^)	0.325 ± 0.019	0.259 ± 0.009*
Adrenal/body weight (×10^5^)	5.95 ± 0.77	5.12 ± 0.33
Perirenal adipose/body weight (×10^2^)	0.429 ± 0.057	0.353 ± 0.032
Heart/body weight (×10^2^)	0.614 ± 0.021	0.634 ± 0.025
Left ventricular free wall thickness/tibial length	0.445 ± 0.017	0.496 ± 0.019*
Septum wall thickness/tibial length	0.512 ± 0.030	0.530 ± 0.021
Right ventricular free wall thickness/tibial length	0.235 ± 0.018	0.253 ± 0.010

* Significantly different between lambs of cortisol-infused ewes as compared to control lambs (*P *<* *0.05).

### Effects of maternal cortisol on neonatal cortisol, ACTH, glucose, and insulin metabolism

Plasma concentrations of ACTH, cortisol, glucose, and lactate were all highest in the samples collected at birth in both groups of lambs (Table 4; Fig. 1G and H, lactate data not shown). There was no overall effect of treatment on the neonatal cortisol concentration. However, both plasma glucose and plasma insulin concentrations were altered by the in utero maternal cortisol treatment. Overall glucose was significantly increased, and plasma insulin was significantly decreased, after maternal cortisol infusion (Fig. 1H and I). This resulted in a significantly reduced insulin to glucose ratio in the neonates of cortisol-treated ewes over the course of the study (control: 48 ± 13; cortisol: 25 ± 6 pmol/mmol).

**Table 4:** Postnatal values of plasma ACTH, renin activity, aldosterone, and creatinine concentrations

	Plasma ACTH (pg/mL)**		Plasma renin activity (ng/mL/h)**		Plasma aldosterone (pg/mL)**		Plasma creatinine (mg/100 mL)**	
	Control	Cortisol	Control	Cortisol	Control	Cortisol	Control	Cortisol
Birth	279 ± 23	304 ± 41	19.8 ± 4.2	10.8 ± 2.5	239 ± 45	233 ± 36	3.7 ± 0.6	2.7 ± 0.3
D3	158 ± 34	162 ± 23	16.5 ± 4.4	12.2 ± 3.5	204 ± 86	123 ± 20	1.1 ± 0.1	1.1 ± 0.1
D6	158 ± 33	246 ± 37	16.6 ± 5.9	10.1 ± 2.6	174 ± 95	110 ± 12	0.9 ± 0.1	1.2 ± 0.2
D9	136 ± 24	208 ± 53	12.7 ± 5.2	10.5 ± 3.3	–	–	1.1 ± 0.2	1.4 ± 0.2
D12	158 ± 22	175 ± ±27	11.8 ± 4.5	9.6 ± 3.7	–	–	–	–
D14	128 ± 14	173 ± 22	12.2 ± 4.1	6.7 ± 2.7	80 ± 14	96 ± 13	1.0 ± 0.1	1.4 ± 0.2*

* Significantly different between groups at this time point.

** Significant effect of postnatal age (*P* < 0.05).

In both glucose challenge studies, the plasma glucose concentration was similar between the two groups of lambs (Fig. 3B and C); however, there was a significant effect of maternal cortisol on the glucose response to glucose injection at 10–12 days of age, when the second glucose challenge test was performed (Fig. 3C). Parameter *a* in the glucose disappearance curve was also significantly higher in the cortisol-treated lambs during both glucose tolerance tests (Table 1); this reflects decreased glucose distribution volume in the offspring of the cortisol-treated ewes. The insulin response to glucose injection at either age was not significantly affected overall by the maternal cortisol infusion (Fig. 3E and F), although in male lambs in the cortisol group, plasma insulin concentrations at 40–90 min were significantly lower than in the male lambs in the control group during the glucose challenge at 10–12 days of age. There was no significant difference between the sexes in the insulin response in either the control or cortisol treatment groups.

**Figure 4:**
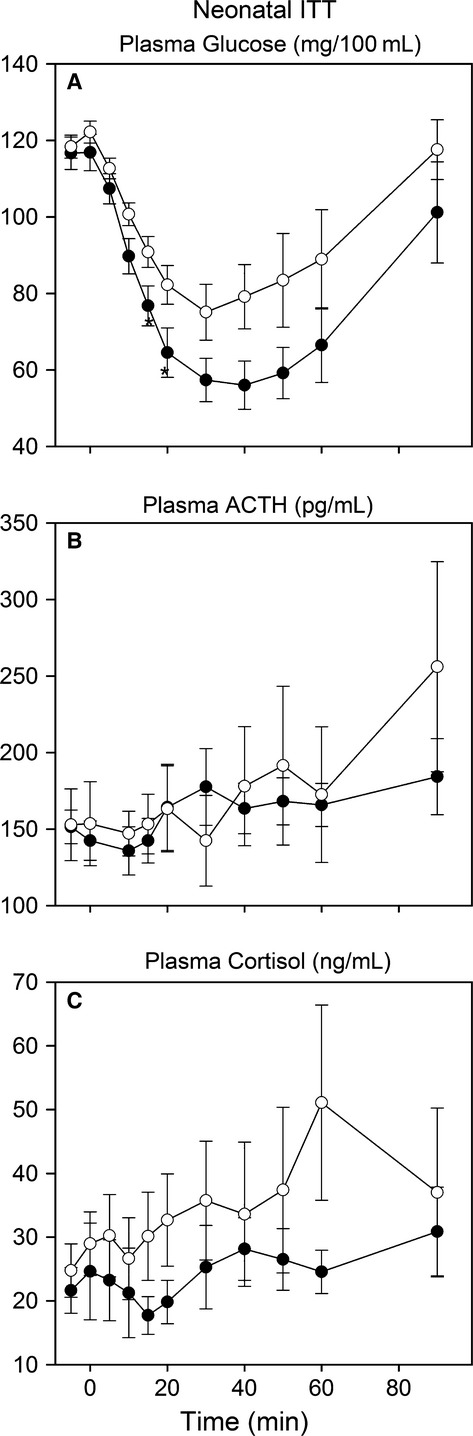
Plasma glucose (A), ACTH (B), and cortisol (C) concentrations in response to intravenous injection of 0.25 U insulin/kg in lambs of control ewes (white circles) and cortisol-infused ewes (black). *Significantly different between groups at this time point.

As a test of HPA axis responsiveness, the ACTH and cortisol responses to a standard insulin tolerance test of 0.25 U insulin per kilogram body weight were determined. As the lambs were not fasted prior to insulin injection, the glucose response to insulin was relatively small; the overall glucose response was not significantly different between the two groups of lambs (*P *=* *0.059), nor was the overall area under the curve for plasma glucose after injection of insulin different between the two groups. However, the cortisol exposed lambs had significantly lower plasma glucose concentrations at 15 and 20 min after the injection of insulin (Fig. 4A), suggesting that insulin sensitivity is increased in the offspring of the cortisol-treated ewes. The ACTH and cortisol responses to this mild hypoglycemic challenge were not different between the two groups of lambs, though the response was highly variable among the individual lambs within each group (Fig. 4B and C).

### Effects of maternal cortisol on neonatal measures of renal function

In both groups of lambs there were significant effects of postnatal age on plasma Na^+^, K^+^, and packed cell volume, with the highest concentrations in the sample collected shortly after birth (Fig. 2K, L, O); conversely, plasma protein and plasma Ca^2+^ significantly increased with postnatal age (Fig. 2M and N). Plasma Na^+^, Ca^2+^, and protein were also significantly altered by maternal cortisol infusions, with greater plasma Na^+^ and lower plasma Ca^2+^ and plasma protein concentrations in the cortisol-exposed lambs (Fig. 2K, M, N). The decrease in packed cell volume was also greater overall in the cortisol-exposed lambs (Fig. 2O). As a result of these changes, we further assessed volume regulation by measuring plasma aldosterone, renin activity, and plasma creatinine concentrations (Table 4). In both groups of lambs, plasma renin activity and plasma aldosterone were highest at birth and significantly decreased over time after birth. Plasma aldosterone concentrations also were not different. In both groups of lambs, plasma creatinine concentrations were higher at birth than on postnatal days 3–14. There was no overall effect of cortisol exposure, although plasma creatinine concentrations were significantly higher in the cortisol-exposed lambs on day 14. There was no effect of sex or the effect of cortisol on creatinine, although female lambs in the control group had significantly higher plasma creatinine at birth than female lambs of cortisol-treated ewes, and overall males in the control group had higher plasma creatinine concentrations than females in the control group.

## Discussion

The results of this study indicate that a modest increase in maternal cortisol concentrations each night during late gestation raises maternal plasma cortisol and alters glucose and insulin regulation in the neonate. This occurs without a chronic increase in maternal glucose or insulin. The overnight increase in maternal cortisol concentrations also appears to alter both left ventricular wall thickness and renal size in the 2-week-old lamb, suggesting there could be longer term effects on both metabolic and cardiovascular health as the neonate matures.

The overnight increase in maternal plasma cortisol had a minor effect on maternal glucose and insulin as compared to our previous study in which cortisol was continuously infused in late gestation (Keller-Wood et al. 2014). Continuous maternal cortisol infusion resulted in chronically elevated maternal glucose and insulin concentrations, and slowed glucose disappearance after administration of a glucose bolus intravenously. In this model, there was a dramatic increase in fetal/neonatal death in the peripartal period. Overnight infusion of cortisol, administering half the daily total dose of cortisol, also resulted in two incidences of unexplained fetal death at term, suggesting that effects on maternal glucose metabolism and on fetal metabolism/survival are cortisol dose related.

The endogenous glucocorticoid, cortisol, has been found to reduce the growth of the axial skeleton in fetal sheep, and the prepartum cortisol surge has been suggested to be responsible for the normal decline in the rate of growth toward the end of gestation (Fowden et al. 1996). Higher doses of glucocorticoids administered to the mother have been found to reduce growth (Johnson et al. 1981), as does maternal Cushing’s syndrome (Lindsay et al. 2005). We hypothesized that chronic maternal cortisol treatment might mimic the effect of Cushing’s syndrome and reduce fetal growth rate; however, this hypothesis was disproved because there was no effect of the maternal hypercortisolemia on body weight at birth or on rate of growth in terms of either neonatal girth or body weight. In a previous study of constant maternal cortisol infusion, the rate of increase in fetal girth was slowed, but the current study suggests that this effect requires either a higher total dose, or constantly increased concentrations throughout the day in order for there to be decreased skeletal growth. Indeed, the change in plasma cortisol expected in the fetus as a result of this infusion of cortisol to the ewe is small relative to the normal increase in cortisol that occurs during the parturient surge in fetal cortisol production. Instead, we found that there was a significant overall effect of cortisol on ponderal index. In particular, ponderal index of the cortisol group was reduced in the males at birth and in the females at day 14. These results indicate that the male and female lambs of the cortisol group are shorter relative to their weight at birth and day 14, respectively, and suggest that body composition may be altered. Although the weight of the perirenal fat pad was not altered, the contribution of subcutaneous and mesenteric fat increases postnatally, and may contribute to differences in body composition. However, fetal cortisol appears to have few stimulatory effects in promoting white adipose tissue growth at birth (Mostyn et al. 2003), although cortisol alters the metabolic function of adipocytes in the adult. In the fetal sheep an intact adrenal is necessary for the late gestational rise in brown adipose tissue activity, and cortisol regulates the concentration of brown adipose tissue-specific UCP1, the mitochondrial protein involved in thermogenesis, allowing for maintenance of core body temperature after birth (Mostyn et al. 2003). The infusion of cortisol in the ewes did not depress the normally high cortisol concentrations in the lambs at birth, and newborn body temperatures were also not altered by the maternal infusion of cortisol.

Although we had hypothesized that increased maternal cortisol concentrations would produce a relative hypoadrenal state in the neonate, there were no significant effects on postnatal ACTH or cortisol concentrations. The maintenance of low fetal cortisol concentrations throughout pregnancy is important for the normal developmental progression of fetal organs, especially the fetal adrenal (Wood and Rudolph 1983). Prior to the surge in cortisol at birth, the fetal HPA is sensitive to feedback signals from increased maternal cortisol (Wood and Rudolph 1984), though at the time of birth the HPA appears to be resistant to cortisol-induced suppression of ACTH (Wood 1987). In humans, antenatal glucocorticoid therapy appears to reduce the HPA responses in the newborn. Infants exposed to antenatal betamethasone treatment had suppressed HPA responses to stressors commonly encountered in the neonatal intensive care unit (Davis et al. 2004; Schäffer et al. 2009). In this study, the 12-h daily infusions of cortisol did not impact adrenal maturation or responsiveness, indicating that higher maternal glucocorticoid concentrations are required for the chronic suppression of adrenal maturation.

Although differences in adrenocortical activity in the adult are known to impact glucose homeostasis, the impact of excess in utero glucocorticoids on neonatal glucose metabolism is not well characterized. Despite similar postnatal cortisol concentrations in the two groups of lambs, neonatal plasma insulin concentrations were significantly decreased with a concomitant increase in neonatal plasma glucose concentrations, resulting in decreased insulin-to-glucose ratio. As compared to their mothers, the neonatal glucose concentrations were relatively high by 3 days of age, and in the control group the plasma insulin concentrations were also higher than in the ewes; in contrast the lambs of cortisol-treated ewes have relatively low insulin concentrations relative to the postnatal glucose concentrations. The active isoform of GR is expressed in humans around the time of islet formation and could modulate development of the pancreas, reducing the number of available β-cells later in life (Phan-Hug et al. 2008). Although islet formation begins on gestational day 24 in the fetal sheep (Cole et al. 2009), islet remodeling continues until after birth in the sheep (at least 10 days of age), as well as in the mouse (to 2–3 weeks of age) and human (to 4–6 months of age) (Titlbach et al. 1985; Kassem et al. 2000; Habener et al. 2005). In vitro studies have demonstrated that glucocorticoids not only reduce islet mass, by decreasing β-cell proliferation and increasing apoptosis, but also reduce insulin content and secretion (Rall et al. 1977; Weinhaus et al. 2000). In the rat, there is a negative correlation between fetal corticosterone concentrations and insulin content, supporting the idea that glucocorticoids have a negative effect on β-cell development (Blondeau et al. 2001). In fact, antenatal glucocorticoid exposure significantly reduces the HOMA-B score in young adults, which is driven by higher glucose concentrations but lower insulin concentrations (Kelly et al. 2012). It is not possible to test for fasting concentrations of glucose or insulin in the lambs without increasing plasma cortisol concentrations, as fasting would require that they be separated from the ewes. The results of the glucose tolerance tests suggest that glucose distribution volume may be decreased in the lambs of cortisol-infused ewes, indicated by the higher *a* parameter in the glucose disappearance curve, which may reflect reduced glucose uptake into tissues. This may also contribute to the small increase in the basal plasma glucose concentrations in these lambs. The insulin responses to the glucose challenges were not different between the groups of neonates. This indicates that the pancreatic response to glucose is not impaired, despite reduced basal values of plasma insulin in the lambs and reduced insulin to glucose ratio at normal glucose concentrations. As these lambs have ready access to milk and are therefore likely to be in a postprandial state, the lower insulin concentrations preglucose challenge suggest that there are decreases in the action of other insulin-stimulatory mechanisms such as gut hormones or increased inhibition by alpha-adrenergic activity. The changes observed in the lambs from this study suggest that there could be long-term consequences to the increase in cortisol during late gestation on glucose disposal and on islet responses to basal, although not hyperglycemia-stimulated, insulin secretion. These effects could contribute to the reduced ponderal index, and may influence long-term nutrient storage.

Neonates from cortisol-exposed ewes had thicker left ventricles of the heart. The normal increase in fetal glucocorticoid production by the ovine and human fetus normally coincides with the maturation of the heart, as cardiomyocytes transition from mononuclear proliferative cells to binucleated terminally differentiated tissue (Jonker et al. 2007; Fowden et al. 1998 ). Late gestation may therefore be a particularly vulnerable period for the fetal heart. Excessive or premature exposure of the fetus to glucocorticoids, either synthetic or naturally occurring, is often associated with alterations in fetal heart development (Rog-Zielinska et al. 2013). Moreover, obstructive hypertrophic cardiomyopathy, with increased thickening of the interventricular septum and left ventricle, has been reported in the newborn of a mother with Cushing’s syndrome (Fayol et al. 2004). In previous studies in the fetus, shorter term chronic maternal cortisol infusion increased the thickness of all three walls of the preterm fetal heart without a significant increase in fetal blood pressure (Jensen et al. 2002; Reini et al. 2008), although longer term infusion until birth did not significantly increase wall thickness later in gestation (Keller-Wood et al. 2014). Higher doses of glucocorticoids increase fetal blood pressure, resulting in hypertrophy of the left ventricle (Tangalakis et al. 1992; Lumbers et al. 2005). As the focus of the present study was metabolic alterations in the newborns, we did not measure blood pressure postnatally in these neonates, and therefore we do not know what role increased blood pressure might play in the wall thickness of these hearts.

The newborns of cortisol-infused ewes had smaller kidneys, and increased plasma sodium concentration. At necropsy plasma creatinine concentrations were also slightly higher in this group, suggesting that there may be physiologic effects, and possibly impaired renal function, that would be evident later in life. Nephrogenesis in the sheep is complete at about 130 days of gestation (Wintour et al. 2003) and glucocorticoid exposure prior to the completion of nephrogenesis in the rat or sheep reduces postnatal nephron number (Ortiz et al. 2003; Wintour et al. 2003; Singh et al. 2007). Glucocorticoids also promote renal tubule cellular differentiation, accelerating functional development and altering the renal sodium handling (Slotkin et al. 1992). Treatment of the pregnant dam with dexamethasone from day 1 of pregnancy until parturition produces pups with reduced kidney weight, glomerular filtration rate, urinary sodium excretion rate, and fractional sodium excretion (Celsi et al. 1998). Similarly, antenatal betamethasone treatment reduces GFR and increases sodium uptake in male, but not female, renal proximal tubule cells, likely through suppression of nitric oxide (Tang et al. 2009; Su et al. 2015). The natural rise in fetal glucocorticoids is associated with an increase in the activity of tubular sodium/hydrogen exchanger (NHE) and (Na^+^K^+^) ATPase during the transition from fetal to neonatal life (Celsi et al. 1991; Guillery et al. 1995). Perinatal dexamethasone administered to the pregnant rat programs an increase in the expression of the Na^+^K^+^2Cl^–^ (NKCC2) and Na^+^Cl^–^ cotransporters in the renal cortex at 2 months, NHE3 in the proximal tubule at 7–8 weeks, and the α1 subunit of (Na^+^K^+^) ATPase from whole kidney at 6 months (Dagan et al. 2007, 2008; Wyrwoll et al. 2007). Thus, the increased in utero exposure to cortisol in this ovine model may have reduced renal maturation, but increased expression of renal sodium transporters, increasing sodium retention in the neonates.

A limitation to this study is that the effects of cortisol on mammary gland characteristics and milk production were not evaluated. Glucocorticoids are essential in establishing the necessary structural components for milk synthesis and secretion, regulating the gene expression of the milk proteins, and prolonging lactation by delaying mammary involution (Casey and Plaut 2007). Since the lambs in this study were reared on ewes, there exists the possibility that milk production and content could have been altered in the ewes exposed to the cortisol treatment, leading to altered neonatal growth and nutrient state. However, as maternal plasma cortisol concentrations were not different in the postpartum period, we do not anticipate differences in milk composition.

Changes in the intrauterine environment have been suggested as a mechanism for programming the offspring to develop diseases that manifest later in life. The changes observed in the neonates of the present study could represent permanent modifications in the mechanisms governing neonatal growth, glucose metabolism, and sodium regulation due to steroid-induced in utero effects. However, what is not clear yet, is whether these changes alter the ability to respond to other stimuli and contribute to the development of adult onset diseases. Further studies are needed in order to evaluate the effects of this model on blood pressure regulation and glucose metabolism in adult life.
